# Trends in the Abscopal Effect After Radiation to Spinal Metastases: A Systematic Review

**DOI:** 10.7759/cureus.5844

**Published:** 2019-10-05

**Authors:** Mohamed Macki, Mohamed Fakih, Jaafar Elmenini, Sharath Kumar Anand, Adam M Robin

**Affiliations:** 1 Neurosurgery, Henry Ford Hospital, Detroit, USA

**Keywords:** abscopal, checkpoint inhibitors, immunomodulators, immunotherapy, metastasis, neurosurgery, radiation, spine, spinal metastases

## Abstract

While the abscopal effect has been previously described, the phenomenon has been poorly defined in the case of spinal metastases. This article is unique in that we present the first systematic review of the abscopal effect after radiation therapy to metastatic spinal cancer, especially since the spinal column represents one of the most common metastatic locations. Following the Preferred Reporting Items for Systematic Reviews and Meta-Analyses (PRISMA) guidelines in the Enhancing the QUAlity and Transparency Of health Research (EQUATOR) resources, a systematic review identified relevant studies via a computer-aided search of MEDLINE and Embase. Ten publications that met the inclusion and exclusion criteria from the PRISMA flow diagram described a total of 13 patients, 76.9% of whom demonstrated image findings of the abscopal effect. In summary, important trends in the nine patients who experienced the abscopal effect in this review include higher doses of radiation and treatment with immunomodulators, both of which may help guide treatment paradigms for spinal metastases superimposed on diffuse metastatic disease. These trends, however, still warrant further investigations with experimental and clinical studies for a mechanistic understanding of the abscopal effect.

## Introduction and background

In 1953, RH Mole first described the abscopal effect as the regression of a tumor remote from the irradiated tissue [1]. The evolution of adjuvant chemotherapies and systemic interventions bolstered a slow rise in the abscopal phenomenon. However, in a paper published in the New England Journal of Medicine in 2012, Postow et al popularized the abscopal effect by describing a case of metastatic melanoma resistant to standard cisplatin, vinblastine, and temozolomide [2]. A trial drug, ipilimumab, concurrent with radiation therapy, actually decreased non-irradiated lesions: right hilar lymphadenopathy and splenic foci. As the second decade of the 21st century ushered in an era of immunotherapies like ipilimumab, there was a rapid upswing in the publication of case reports and case series with encouraging results with the abscopal effect [3]. These immunomodulators, more specifically called “checkpoint inhibitors,” promised a synergistic phenomenon with radiation therapy. But, even though the spinal column represents one of the most common sites of metastases, the abscopal effect in the context of spinal oncology has been poorly described. We present the first systematic review on the abscopal effect after radiation to spinal metastases. The objective of this systematic review is to identify unique features that may increase abscopal successes after irradiating spinal lesions.

## Review

Methods

**Figure 1:**
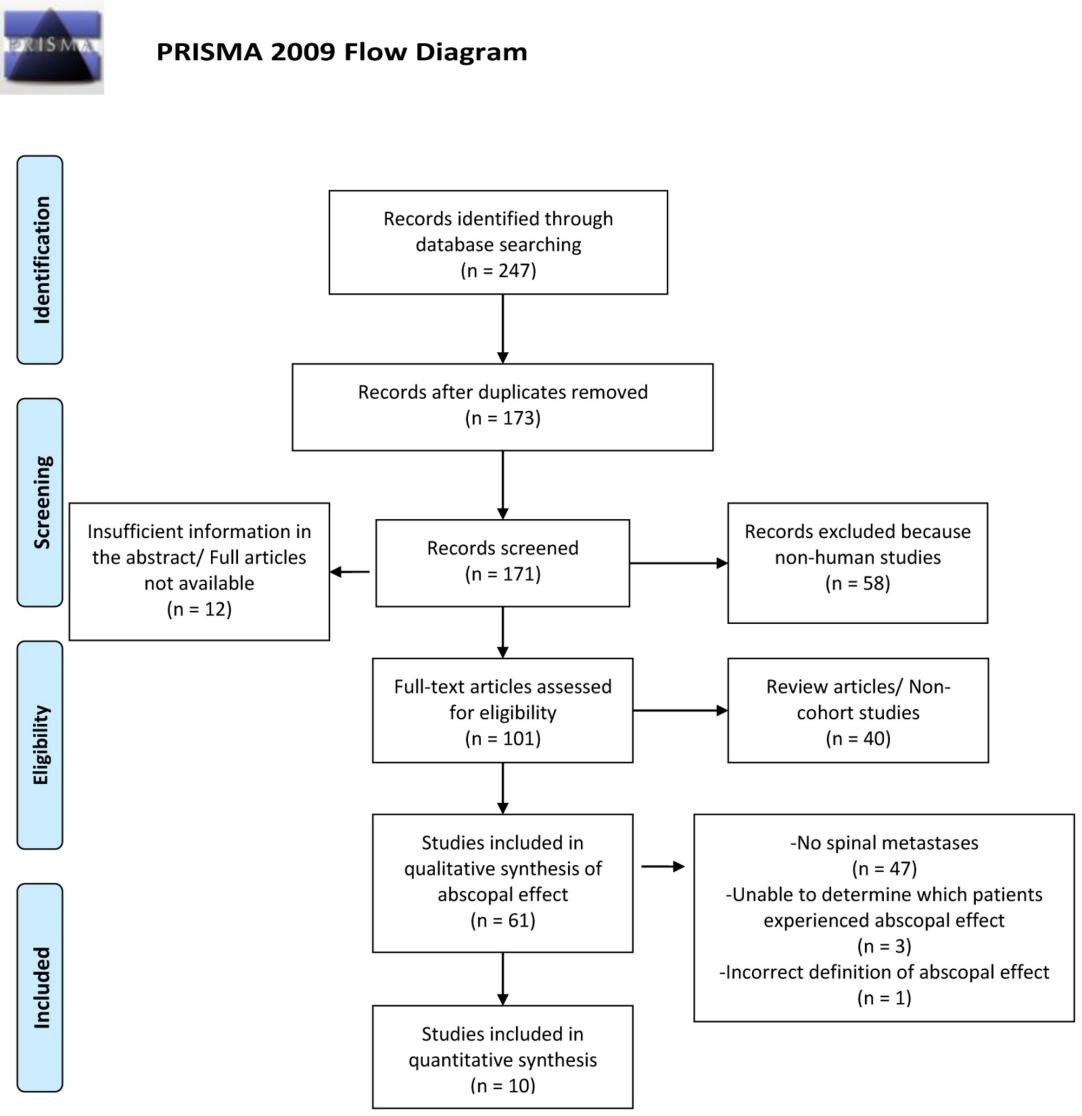
PRISMA Flow Diagram From:  Moher D, Liberati A, Tetzlaff J, Altman DG, The PRISMA Group (2009) [4]

Following the Preferred Reporting Items for Systematic Reviews and Meta-Analyses (PRISMA) guidelines in the Enhancing the QUAlity and Transparency Of health Research (EQUATOR) resources, a systematic review identified relevant studies via a computer-aided search of MEDLINE (1946 - October 18, 2018) and Embase (1947 - October 18, 2018) (Figure 1) [4]. Articles were extracted following our institution's Library Protocol for Systematic Reviews, which provides a systematic and reproducible scan of articles in MEDLINE, Embase, and Cochrane. Per this protocol, all citations were collected by a trained reference analyst with a Master of Library and Information Science (MLIS) degree and who is a designee by the Academy of Health Information Professionals (AHIP). No specific automated search software was utilized. Key words include “abscopal” AND “spine” OR “spinal” OR “bone” OR “cervical” OR “thoracic” OR “lumbar” OR “sacral” OR “sacrum” OR “coccyx” OR “vertebral” OR “vertebrae” OR “bony” OR “lumbosacral” OR “thoracolumbar” OR “bone neoplasms” OR “spine neoplasms” and derivatives thereof. The references within literature reviews and systematic reviews generated by the computer-aided search were also scrutinized for relevant studies. Non-English publications were excluded from the search strategy. Unpublished studies were not identified from MEDLINE, Embase, and Cochrane. Published abstract presentations, on the other hand, were included in the search paradigm.

Following removal of duplicated studies, articles were screened for non-human studies. Abstracts without full texts available were removed if information was insufficient to complete the columns in Table 1. Published abstracts from conferences were traced by searching for the first author and a few key words in Google Scholar, which occasionally helped us identify the original article. Next, full-text articles were evaluated, and review articles and non-cohort studies were excluded. Lastly, patients without spinal metastases or hematological cancers were not included. This PRISMA paradigm selected for specified articles: (1) cases with metastatic and/or hematological cancer to the spine; AND (2) “abscopal” in the title, abstract, and/or keywords. Data were extracted from the articles by two independent reviewers.

While the abscopal effect may not have been explicitly defined in the articles summarized in Table 1, all authors did unanimously report the phenomenon as a regression of metastatic tumor burden outside of the radiated field. One case report illustrated an abscopal effect as an adverse reaction distant to the radiated target volume [5]. The citation was removed due to an “incorrect definition of abscopal effect” in Figure 1.

Results

Ten articles discussed the abscopal effect after radiation therapy to metastatic spinal lesions. These ten publications that met the inclusion and exclusion criteria from the PRISMA flow diagram described a total of 13 patients in Table 1 [6-16]. Of the nine cases specifying age, 59.4 years was the average. Of the nine cases identifying gender, four (44.4%) were males. Tumor pathology included four melanoma (30.7%), two renal carcinoma (15.3%), two invasive ductal breast carcinoma (15.3%), one hepatocellular carcinoma (7.6%), one Hodgkin’s lymphoma (7.6%), one squamous cell lung cancer (7.6%), one urothelial bladder cancer (7.6%), and one endometrial adenocarcinoma (7.6%). Of the total 13 patients in 10 publications, nine patients (76.9%) demonstrated image findings of the abscopal effect.

**Table 1:** Systematic Review Publications commenting on the abscopal effect after metastatic cancer to the spine

Author	Number of Patients	Age	Sex	Primary	Metastatic Areas	Systemic Therapy	Radiation Therapy	Surgical Resection	Abscopal Effect
Ohba et al, 1998 [14]	1	76	M	Hepatocellular carcinoma	T2 vertebrae; new, recurrent hepatic lesions	Mitomycin, epirubicin, doxorubicin	Thoracic vertebral bone lesion: total dose of 36 Gy	Partial resection of affected liver	Remarkable regression of the hepatic lesions to very small masses
Ishiyama et al, 2012 [10]	1	61	M	Renal cell carcinoma, clear type	Adrenal gland; bilateral lung nodules, and multiple mediastinal and hilar lymphadenopathy; lytic bone lesions in posterior acetabulum and in T8 and T10 vertebral bodies; brain metastases	No systemic therapy	Brain metastases: stereotactic radiosurgery (SRS) to dose of 18 Gy; bone and spinal lesions: stereotactic body radiation therapy (SBRT) dose of 40 Gy in five fractions	Left nephrectomy	Almost complete disappearance of untreated multiple lung metastases and lymphadenopathy; relapse of brain lesions
Grimaldi et al, 2014 [7, 8]	Only 2/21 patients were treated with spinal radiation	N/A	N/A	Melanoma	Lung and vertebral metastases	Ipilimumab	Vertebral radiation therapy: 30Gy/10 fractions	None	Abscopal effect to the lung lesion [11/21 patients in the index study population experienced an abscopal effect]
Grimaldi et al, 2014 [7, 8]	Only 2/21 patients were treated with spinal radiation	N/A	N/A	Melanoma	Vertebral metastases; other metastatic foci unknown	Ipilimumab	N/A	None	No abscopal effect
Hardy et al, 2015 [9]	Only 1/10 patients were treated with spinal radiation	25	F	Hodgkin’s lymphoma	Lumbar 3-5	Donor lymphocyte infusion (DLI)	8 Gy radiation therapy	none	Systemic immune responses suggested by T-cell proliferation in the peripheral blood as well as upregulation of interferon (IFN)-inducible genes and tissue damage receptors in non-irradiated tumor [10/ 10 patients in the index study population experienced an abscopal effect]
Levy et al, 2016 [12]	Only 2/10 patients were treated with bony radiation	65	M	Squamous cell lung carcinoma	Lymph node, bone, and liver	The entire study population of 10 patients were treated with durvalumab	Spine C7-T4, T7-T10, L5-S1, L2: 28Gy/5 fractions; Iliac bone: 36 Gy/10 fractions; lung and liver treatment regimens cannot be specified	None	No abscopal effect was observed in the entire study population of 10 patients
Levy et al, 2016 [12]	Only 2/10 patients were treated with bony radiation	58	F	Urothelial bladder carcinoma	Lymph node, bone, lung, and liver	The entire study population of 10 patients were treated with durvalumab	Spine C7-T4, T7-T10, L5-S1, L2: 28Gy/5 fractions; iliac bone: 36 Gy/10 fractions; lung and liver treatment regimens cannot be specified	None	No abscopal effect was observed in the entire study population of 10 patients
Ribeiro et al, 2016 [15]	Only 2/16 patients were treated with spinal radiation	54	M	Both with melanoma [study population: 12 melanoma; 2 non-small cell lung cancer; 2 renal cell carcinoma]	Lung; cervical spine	Ipilimumab then nivolumab	For the study population of 16 patients, the median total dose was 24Gy (1–40Gy), and the doses were, in general, given in 3 fractions (1–10 fractions)	None	Abscopal effect to non-irradiated pulmonary nodules [3/16 patients in the index study population experienced an abscopal effect, all of which with melanoma primary]
Ribeiro et al, 2016 [15]	Only 2/16 patients were treated with spinal radiation	N/A	N/A	Both with melanoma [study population: 12 melanoma; 2 non-small cell lung cancer; 2 renal cell carcinoma]	- Vertebrae	Anti-PD1 inhibitor unspecified	For the study population of 16 patients, the median total dose was 24Gy (1–40Gy), and the doses were, in general, given in 3 fractions (1–10 fractions)	N/A	No abscopal effect
LaPlant et al, 2017 [11]	1	N/A	N/A	Renal cell adenocarcinoma	Lungs; thoracic lymph nodes; sacrum	Ipilimumab and nivolumab	Dose-painting stereotactic body radiation therapy: 18 Gy to the periphery of the tumor and 27 Gy to the center of the lesion over three fractions concurrent with immunomodulators	Pelvic mass excision	No evidence of pulmonary or nodal metastases and unchanged residual treated tissue in the sacrum
Leung et al, 2018 [16]	1	65	F	Invasive ductal carcinoma	T8; axillary lymph nodes	None	Breast: 225 Gy/15 fractions; thoracic spine: 50 Gy/25 fractions	None	Remarkable reduction in the axillary lymph nodes
Oh et al, 2018 [13]	1	64	F	Endometrial adenocarcinoma	Left thigh cutaneous metastases; pelvic and para-aortic lymphadenopathy; L3 spinal metastasis; bladder mass; positive nodes in the retroperitoneum, external iliacs, and left inguinal chain	Nivolumab	Dosing not specified	Total abdominal hysterectomy	Strong partial response not only in the targeted lesions but also throughout metastatic tumor burden
Azami et al, 2018 [6]	1	67	F	Invasive ductal breast carcinoma	Lung metastasis; femur, lumbar vertebrae and sacrum; positive lymph nodes: in lung, right axilla, right supraclavicular area and the mediastinum	Anastozole	Right breast: 60 Gy; left femur: 28 Gy; lumbar vertebrae and sacrum: 39 Gy (daily 2-Gy fractionated dose)	None	Complete remission in all sites exhibiting ^18^F-fluorodeoxyglucose (FDG) uptake on positron emission tomography (PET)

In 2012, Ishiyama et al and Leung et al represented the only case reports that did not include any systemic therapies, yet the abscopal effect was still reported following radiation therapy to the spine [10, 16]. All remaining eight articles commented on the abscopal effect in the setting of both systemic therapy and radiation therapy. In the formerly mentioned intervention, adjuvant anti-neoplastic medications at first focused on chemotherapies (mitomycin, epirubicin, doxorubicin) with the 1998 publication by Ohba et al [14]. Azami et al also reported a similar phenomenon with anastozole [6]. Not until 2014 did Grimaldi et al report the abscopal effect after an immunomodulator - in this case, the anti-CTLA-4 (cytotoxic T-lymphocyte-associated protein 4) antibody, ipilimumab [7, 8]. Yet, in 2016, Levy et al failed to observe any abscopal effects with the anti-PD-L1 (programmed death-ligand 1) antibody, durvalumab [12]. In that same year, Ribeiro et al illustrated an abscopal effect with ipilimumab followed by the anti-PD-1 (programmed cell death protein 1) antibody, nivolumab [15]. LaPlant et al and Oh et al similarly described distant regression of metastatic disease with anti-PD-1 inhibitors [11, 13]. Thus, four of the nine patients who experienced the abscopal affect were treated with immunomodulators. Lastly, in the 2015 publication on advanced-stage Hodgkin’s lymphoma, Hardy et al was the sole reference of the abscopal affect with radiation to the lumbar spine following a transfusion of donor lymphocytes without specifying any chemotherapeutic or immunomodulating medications [9].

All articles in Table 1 included radiation therapy to the spine. Target fields include cervical spine in two publications, thoracic spine in four publications, lumbosacral spine in four publications, low sacrum in one publication, and an unspecified spinal region in one publication. Radiation dose to the spine in Gray (Gy) per fraction of therapy were specified in eight publications (listed in descending order of Gy/fraction): 18 Gy to the periphery of the tumor and 27 Gy to the center of the lesion over three fractions for renal adenocarcinoma [11], 8 Gy per fraction (40 Gy/5 fractions) for renal clear cell carcinoma [10], 8 Gy in one fraction for Hodgkin’s lymphoma [9], approximately 6 Gy per fraction (28 Gy/5 fractions) for squamous cell lung carcinoma and urothelial bladder carcinoma [12], 3 Gy per fraction (30 Gy/10 fractions) for melanoma [7, 8], 2 Gy per fraction (50 Gy/25 fractions) for invasive ductal carcinoma [16], 2 Gy/fraction (39 Gy with a daily 2 Gy fractionated dose) for invasive ductal breast carcinoma [6], and an average of 24 Gy (range 1-40 Gy) delivered over an average of three fractions (range: 1-10 fractions) for melanoma [15]. Ohba et al reported a total of 36 Gy without specified fractions for hepatocellular carcinoma, and dosing for endometrial adenocarcinoma was not specified by Oh et al [13, 14]. Notice, of the nine patients who experienced the abscopal effect, four were associated with higher doses of radiation therapy to the metastatic spinal disease: 6-9 Gy/fraction in renal adenocarcinoma [11], 8 Gy/fraction in renal clear cell carcinoma [10], 8 Gy/fraction in Hodgkin’s lymphoma [9], and and an average of 24 Gy (range 1-40 Gy) delivered over an average of three fractions (range: 1-10 fractions) for melanoma [15].

Three authors failed to observe an abscopal effect in four patients. While Grimaldi et al noted regression of the distant lung lesion after spinal radiation in one patient, a second patient with metastatic melanoma treated with ipilimumab did not experience an abscopal phenomenon [7, 8]. In total, 11 of the 21 patients in their index study population had an abscopal effect. Next, Levy et al could not appreciate the phenomenon in any of their 10 patients treated with durvalumab and spinal radiation therapy (approximately 6 Gy/ fraction) [12]. Although Ribeiro et al reported a decrease in non-irradiated lung nodules after spinal radiation in one patient, a similar case of metastatic melanoma treated with ipilimumab then nivolumab did not experience an abscopal effect [15]. In total, 3 of their 16 patients with melanoma, non-small cell lung cancer, or renal cell carcinoma had the phenomenon.

Discusssion

Although regression of lesions distant to the radiation site has been reported since the 1950s, the phenomenon has been sparingly described over the ensuing half-a-century. Only 46 reported cases of the abscopal effect were published within the 31 articles of a systematic review by Abuodeh et al in 2014 [17]. Within the past several years, however, the advent of immunotherapies marks a renaissance of the abscopal effect with the management of metastatic disease. In 2011, the Food and Drug Administration (FDA) approved one of the first immunomodulators: the anti-CTLA-4 monoclonal antibody, ipilimumab [18, 19]. The subsequent years in this first systematic review of the abscopal effect after radiation therapy to metastatic spinal disease saw a rise in case reports and case series on abscopal observations with the dawn of immunomodulators. Prior to that time, the abscopal effect was sparingly described without any systemic therapies [10, 16], with chemotherapy [6, 14], or with donor lymphocyte infusion [9].

Cancer biologists have determined that a diverse repertoire of receptors on intratumoral T-lymphocytes increases the attack on neoplastic cells [20, 21]. But this yields only a modest increase in the number of CD8+ T-cells (cytotoxic lymphocytes). Enhancing this response, the anti-CTLA-4 inhibitors (e.g., ipilimumab, tremelimumab) block the CTLA-4 receptor on regulatory T-cells (Treg, a derivative of CD4+ T-cells that inhibit other white blood cells via IL-10 and TGF-β) in order to increase the CD8+ T-cell: Treg ratio. An understanding of the proportional relationship between these T-cell subtypes has laid the foundation for developing other classes of immunotherapies, i.e., the inhibitors of programmed cell death. Evidently, a decline in the critical T-cell ratio indicates that cytotoxic lymphocytes are becoming overwhelmed by uncontrolled replication of the neoplasm. These “exhausted T-cells” uniquely express the PD-L1 receptor, PD-1, and the transcription factor Eomes, which signal for apoptosis of a non-functioning lymphocyte. By inhibiting this downward signaling cascade, the programmed cell death inhibitors, anti-PD-1 and anti-PD-L1 antibodies, encourage oligoclonal CD8+ T-cell expansion and prevent the resistance of cytotoxic lymphocytes against tumor cells.

In 2015, a publication in Nature demonstrated that these immune checkpoint inhibitors in conjunction with radiation bolstered the immune-mediated response for superior control of tumor burden [22]. The interplay between radiation therapy and these novel drugs underlies the mechanism of the abscopal effect [20]. The irradiated tumor releases neoantigens, known as tumor-associated antigens (TAAs), among the apoptotic and necrotic tumor cells [23]. The TAAs may be engulfed by antigen-presenting cells (APCs) for presentation to CD8+ T-cells, which can subsequently attack not only the primary tumor but also the metastatic disease. Another mechanism focuses on the release of danger-associated molecular patterns (DAMPs) from the irradiated tumor cells [24]. The DAMPs bind to pattern recognition receptors, for example, toll-like receptors (TLR), that stimulate a systemic pro-inflammatory response. Regardless of the inciting antigen, the capacity for checkpoint inhibitors to increase the CD8+ T-cell:Treg ratio invariably strengthens the cytotoxic lymphocyte’s response to TAAs and DAMPs. Moreover, in the review on immunotherapy to boost the abscopal effect, Ngwa et al found in preclinical trials that substantial abscopal responses occur when immunotherapies follow (or at least simultaneously administered with) radiotherapy [20]. While the exact reasoning has not been completely described, a compelling theory suggests that initial surgery and/or radiotherapy tend to debulk the tumor burden and, subsequently, circumvent T-cell exhaustion. The association between the timing of immunotherapy and cancer response is particularly important in spinal metastases. In this systematic review, systematic therapies in all eight articles were administered in conjunction with or following radiation therapy. In a retrospective review of metastatic melanoma treated with spinal decompression and fusion and radiation, patients preoperatively treated with immunotherapies had a median survival of 98 days that was statistically significantly lower than the median of 315 days in patients not on those drugs preoperatively [25]. Again, this substantiates claims that immunomodulators should not precede tumor debulking with surgery and/or radiation.

The review by Ngwa et al also concluded that the abscopal effects were associated with higher Gy per fraction of radiation [20]. In the animal models comparing fractionated to single-dose radiotherapy in the immune-mediated abscopal effect, Dewan et al discovered that the regimen of 8 Gy in three fractions was superior to both 20 Gy in one fraction and 6 Gy in five fractions for the induction of tumor-specific T-cells and, thereby, the abscopal effect [26]. Morisada et al noted that in mice treated with high-dose, hypofractionated irradiation, as compared to low-dose hyperfractionated therapies, enhances anti-tumoral immunity measured by “tumor microenvironment” and “tumour-draining lymph nodes” tumor-specific T-lymphocytes [27]. When combined with PD-1 monoclonal antibodies intended to reverse adaptive immune resistance, distant tumors in the mice models also regressed. Similarly, in this systematic review, all publications citing a positive abscopal effect utilized a fractionated dose of radiation therapy, except for the case of 8 Gy/1 fraction to the lumbar spine for Hodgkin’s lymphoma [9]. The balance between single dose and hyper-fractionated dosing of radiation has not been completely elicited. In the case presentation on abscopal effect after irradiation to the T8 spine without adjuvant systemic therapies or surgery in Table 1, Leung et al concluded, “The tumor immunity induced by radiation therapy may be influenced by the radiation dose and fractionation” [16]. Another report argues that the ideal situation likely reflects a higher number of Gy in a select few fractions, just enough to induce interferon (IFN)-related genes, such as activators of transcription and, thus, signal transducers [28].

Lastly, abscopal observations may have a higher propensity for success with radiation to the spinal column over other metastatic areas. Hematopoietic stem cells residing in the bone marrow throughout the spine are particularly amenable to direct ionizing radiation that elicits an innate immune recognition of tumor even in the absence of tumor antigens via the release of “danger signals” - i.e., cellular stress signals [29]. This becomes most evident in hematological malignancies wherein irradiation of the reticuloendothelial system triggers a decrease in circulating malignant blood cells, explained by a cytotoxic effect on cancerous cells circulating through the radiated area [29-31]. In case of Hodgkin’s lymphoma within this systematic review, Hardy et al reported a similar phenomenon with the donor lymphocyte infusion leading to an abscopal effect, defined as T-cell proliferation in the peripheral blood as well as upregulation of IFN-inducible genes and tissue damage receptors in a non-irradiated tumor [9]. Rees et al reported a similar abscopal observation in 10 cases out of 895 patients with Hodgkin’s or non-Hodgkin’s lymphoma treated with radiation therapy [32]. Learning from these treatment paradigms in hematological malignancies, such mechanisms for ionizing bone marrow that naturally harbors lymphocyte populations may be extrapolated to the abscopal success with radiating spinal metastases. The irradiated hematopoietic stem cells living in the bone marrow may appropriately differentiate lymphocytes, circulate throughout the body, and attack distant metastatic foci. While compelling, these hypotheses require further experiential and clinical testing.

## Conclusions

This article is unique in that we present the first systematic review of the abscopal effect after radiation therapy to metastatic spinal cancer. Important trends in the nine patients who experienced the abscopal effect in this review include higher doses of radiation and treatment with immunomodulators, both of which may help guide treatment paradigms for spinal metastases superimposed on diffuse metastatic disease. These trends, however, still warrant further investigations with experimental and clinical studies for a mechanistic understanding of the absopal effect.

## References

[1] Mole RH (1953). Whole body irradiation; radiobiology or medicine?. Br J Radiol.

[2] Postow MA, Callahan MK, Barker CA (2012). Immunologic correlates of the abscopal effect in a patient with melanoma. N Engl J Med.

[3] Luke JJ, Lemons JM, Karrison TG (2018). Safety and clinical activity of pembrolizumab and multisite stereotactic body radiotherapy in patients with advanced solid tumors. J Clin Oncol.

[4] Moher D, Liberati A, Tetzlaff J (2009). Preferred reporting items for systematic reviews and meta-analyses: the PRISMA statement. PLoS Med.

[5] Shueng PW, Lin SC, Chang HT (2009). Toxicity risk of non-target organs at risk receiving low-dose radiation: a case report. Radiat Oncol.

[6] Azami A, Suzuki N, Azami Y (2018). Abscopal effect following radiation monotherapy in breast cancer: a case report. Mol Clin Oncol.

[7] Grimaldi AM, Simeone E, Giannarelli D (2014). Abscopal effects of radiotherapy on advanced melanoma patients who progressed after ipilimumab immunotherapy. Oncoimmunology.

[8] Grimaldi AM, Simeone E, Giannarelli D (2013). The abscopal effect: efficacy of radiotherapy in patients on progression after treatment with ipilimumab 3 mg/kg. JITC.

[9] Hardy NM, Citrin D, Hakim FT (2015). Pilot study of radiation-targeted donor lymphocyte infusion for cancer progression after allogeneic hematopoietic stem cell transplantation. Blood.

[10] Ishiyama H, Teh BS, Ren H (2012). Spontaneous regression of thoracic metastases while progression of brain metastases after stereotactic radiosurgery and stereotactic body radiotherapy for metastatic renal cell carcinoma: Abscopal effect prevented by the blood-brain barrier?. Clin Genitourin Cancer.

[11] LaPlant Q, Deselm C, Lockney NA, Hsieh J, Yamada Y (2017). Potential abscopal response to dual checkpoint blockade in rcc after reirradiation using dose-painting sbrt. Pract Radiat Oncol.

[12] Levy A, Massard C, Soria JC, Deutsch E (2016). Concurrent irradiation with the anti-programmed cell death ligand-1 immune checkpoint blocker durvalumab: single centre subset analysis from a phase 1/2 trial. Eur J Cancer.

[13] Oh MS, Chae Y (2018). Repeated abscopal effect with radiation and anti-pd-1 blockade in MMR deficient endometrial cancer. AACR.

[14] Ohba K, Omagari K, Nakamura T (1998). Abscopal regression of hepatocellular carcinoma after radiotherapy for bone metastasis. Gut.

[15] Ribeiro Gomes J, Schmerling RA, Haddad CK (2016). Analysis of the abscopal effect with anti-pd1 therapy in patients with metastatic solid tumors. J Immunother.

[16] Leung HW, Wang SY, Jin-Jhih H (2018). Abscopal effect of radiation on bone metastases of breast cancer: a case report. Cancer Biol Ther.

[17] Abuodeh Y, Venkat P, Kim S (2016). Systematic review of case reports on the abscopal effect. Curr Probl Cancer.

[18] Robert C, Thomas L, Bondarenko I (2011). Ipilimumab plus dacarbazine for previously untreated metastatic melanoma. N Engl J Med.

[19] Hodi FS, O'Day SJ, McDermott DF (2010). Improved survival with ipilimumab in patients with metastatic melanoma. N Engl J Med.

[20] Ngwa W, Irabor OC, Schoenfeld JD (2018). Using immunotherapy to boost the abscopal effect. Nat Rev Cancer.

[21] Stone HB, Peters LJ, Milas L (1979). Effect of host immune capability on radiocurability and subsequent transplantability of a murine fibrosarcoma. J Natl Cancer Inst.

[22] Twyman-Saint Victor C, Rech AJ, Maity A (2015). Radiation and dual checkpoint blockade activate non-redundant immune mechanisms in cancer. Nature.

[23] Grass GD, Krishna N, Kim S (2016). The immune mechanisms of abscopal effect in radiation therapy. Curr Probl Cancer.

[24] Barker HE, Paget JT, Khan AA (2015). The tumour microenvironment after radiotherapy: mechanisms of resistance and recurrence. Nat Rev Cancer.

[25] Shankar GM, Choi BD, Grannan BL (2017). Effect of immunotherapy status on outcomes in patients with metastatic melanoma to the spine. Spine (Phila Pa 1976).

[26] Dewan MZ, Galloway AE, Kawashima N (2009). Fractionated but not single-dose radiotherapy induces an immune-mediated abscopal effect when combined with anti-ctla-4 antibody. Clin Cancer Res.

[27] Morisada M, Clavijo PE, Moore E (2018). Pd-1 blockade reverses adaptive immune resistance induced by high-dose hypofractionated but not low-dose daily fractionated radiation. Oncoimmunology.

[28] Tsai MH, Cook JA, Chandramouli GV (2007). Gene expression profiling of breast, prostate, and glioma cells following single versus fractionated doses of radiation. Cancer Res.

[29] Siva S, MacManus MP, Martin RF (2015). Abscopal effects of radiation therapy: a clinical review for the radiobiologist. Cancer Lett.

[30] Byhardt RW, Brace KC, Wiernik PH (1975). The role of splenic irradiation in chronic lymphocytic leukemia. Cancer.

[31] Li JG (1963). The leukocytopenic effect of focal splenic x-irradiation in leukemic patients. Radiology.

[32] Rees GJ (1981). Abscopal regression in lymphoma: a mechanism in common with total body irradiation?. Clin Radiol.

